# Pleural cryptococcosis diagnosed by pleural biopsy in an immunocompromised patient: a case report

**DOI:** 10.3389/fcimb.2023.1258021

**Published:** 2023-10-25

**Authors:** Hongxia Jia, Hongping Liu, Meng Tu, Yan Wang, Xudong He, Guojun Zhang

**Affiliations:** Department of Pulmonary and Critical Care Medicine, the First Affiliated Hospital of Zhengzhou University, Zhengzhou, China

**Keywords:** pleural cryptococcosis, pleural effusion, thymoma, immunocompromised, pleural biopsy

## Abstract

**Objective:**

The aim of this study is to report an isolated pleural cryptococcosis with pleural effusion as the only manifestation, confirmed by pleural biopsy in a patient with thymoma combined with myasthenia gravis, who developed pleural effusion of unknown origin after long-term glucocorticoids and tacrolimus therapy.

**Methods:**

Pathological examination of the right pleural biopsy tissue from a patient with unexplained recurrent pleural effusion was implemented. Morphological analysis of the fungal component and metagenomic next-generation sequencing (mNGS) on the pleural tissue were performed.

**Results:**

A biopsy specimen of the right pleura revealed numerous yeast-like organisms surrounded by mucous capsules and *Cryptococcus* neoformans was detected by mNGS with a species-specific read number (SSRN) of 4, confirming the diagnosis of pleural cryptococcosis. Pleural effusion was eliminated with amphotericin B and fluconazole, and healthy status was maintained at the time of review 1 year later.

**Conclusion:**

Cryptococcosis, manifested by simple pleural effusion, is extremely rare, but when repeated pleural effusion occurs in immunocompromised patients or in patients with malignant tumors, the possibility of cryptococcosis should be treated with high vigilance and pleural biopsy is recommended if necessary in order to confirm the diagnosis.

## Introduction

*Cryptococcus* has gone from an environmental saprophyte to a global pathogen (May et al., 2016). Cryptococcosis is an opportunistic infection, caused predominantly by *Cryptococcus neoformans* or *Cryptococcus gattii*, which mainly affects immunocompromised patients. The most common manifestation of cryptococcosis is meningitis. Pulmonary involvement is less common than nervous system involvement (RM et al., 2005). Computerized tomography (CT) findings of pulmonary lesions caused by *Cryptococcus* infection can present as nodules, consolidation, and cavities, while pleural effusion rarely occurs cryptococcal (Newman et al., 1987). Isolated pleural cryptococcosis is extremely rare. Here, we report an isolated pleural cryptococcosis with bilateral pleural effusion as the only manifestation, confirmed by pleural biopsy in a patient with thymoma.

## Case presentation

**Figure 1:**
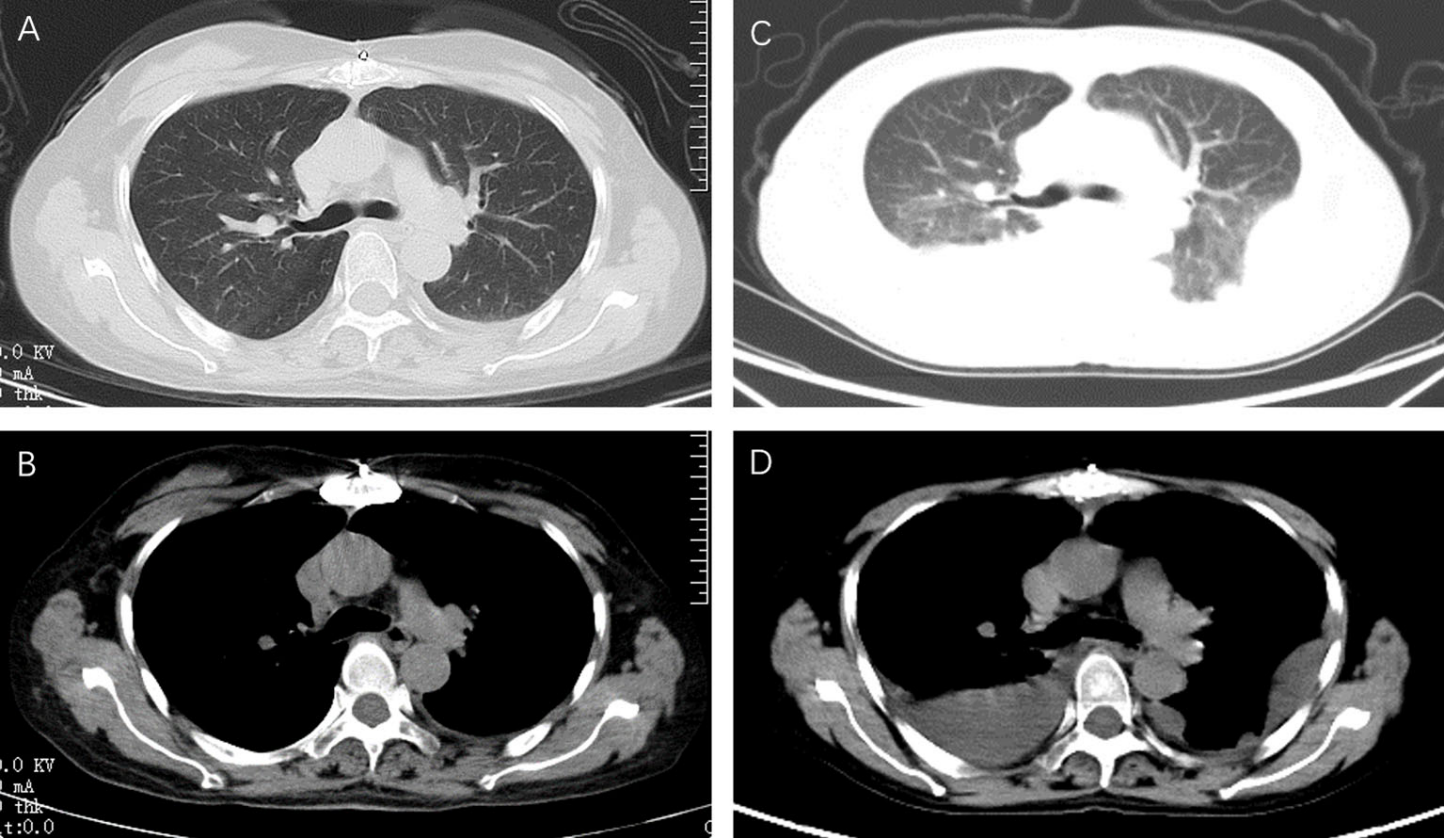
Chest computed tomography at different time points. In the patient with myasthenia gravis after thymoma surgery, a small left pleural effusion was noted on a transverse axial chest CT during regular follow-up **(A, B)**. According to all examinations, the neurologist thought that the chest tightness was due to the recurrence of myasthenia gravis. Methylprednisolone (40mg per day) combined with gamma globulin (400mg per kg) was given. After three days of treatment, the patient's dyspnea worsened. A chest CT was taken again, showed a new bilateral pleural effusion **(C, D)**.

The patient was a 49-year-old woman who was admitted to the hospital on 9 November 2021 with a 2-day history of chest tightness and chest pain. Six months ago, she presented with dyspnea and was diagnosed with myasthenia gravis combined with thymoma. After surgical resection of the thymoma, she was treated with methylprednisolone tablets (60 mg once daily, minus 4 mg monthly), tacrolimus capsules (10 mg twice daily), and pyridostigmine bromide tablets (60 mg twice daily) for 6 months. Five days before admission, she had a chest CT that had a little pleural effusion on the left side (**Figure 1**). Combined with her medical history, she was admitted to the neurology department for treatment of myasthenia gravis. At admission, laboratory tests revealed a serum albumin concentration of 30.6 g/L (reference range, 35 to 55 g/L) and a brain natriuretic peptide level of 208.6 pg/mL (reference range, 0 to 121 pg/mL). The count of CD4+ T cell count was 197.25 cells/μL (reference range, 550 to 1,440 cells/μL). There were no abnormalities in liver function, renal function, examination of connective tissue disease, serum 1, 3-β-D glucan test (G test, reference range, 0 to 95 pg/mL), galactomannan test (GM test, reference range, 0 to 0.5 μg/L), C- reactive protein (CRP, reference range, 0 to 5 mg/L), and procalcitonin (PCT, reference range, 0 to 0.046 ng/mL).

## Initial treatment

According to the above examination, the neurologist thought that the chest tightness was due to the recurrence of myasthenia gravis. Methylprednisolone (40 mg/day) combined with gamma globulin (400 mg/kg) was given. After 3 days of treatment, the patient’s dyspnea worsened. A chest CT was taken again and showed a new bilateral pleural effusion (**Figure 1**). Laboratory tests showed a serum albumin concentration of 28.7 g/L. Albumin supplements in combination with diuretics were given because it was thought that bilateral pleural effusions were associated with low serum albumin. However, the patient’s dyspnea was not relieved, and pleural ultrasound showed that there was still a moderate amount of pleural effusion.

## Bilateral pleural effusion

A closed drainage of the bilateral thoracic cavity was performed on the patient. Approximately 300 ml of pleural fluid was drained per day on each side. The left pleural fluid was a pale yellow exudate with the following laboratory results: protein 44.1 g/L (reference range, 0 to 30 g/L), glucose 4.71 mmol/L (reference range, 3.6 to 5.5 mmol/L), LDH 206 U/L (reference range, 75 to 245 U/L), and cell count 2,223×10^6^/L (61.3% multinucleated cells). The right pleural fluid was also a pale yellow exudate with the following laboratory results: protein 45.1 g/L, glucose 8.23 mmol/L, LDH 147 U/L, and cell count 1,420×10^6^/L (58.4% multinucleated cells) (**Table 1**). We tested for tuberculosis and malignancy as possible causes of the pleural effusion because of the predominance of multiple-nuclear cells. The adenosine deaminase (ADA) level in the left fluid was 9 IU/L and 11 IU/L in the right (reference range, 0 to 22 U/L), and a polymerase chain reaction (PCR) analysis of the effusion for tuberculosis showed negative results. In addition, the tumor markers were all within normal range, and cultures of the bilateral pleural fluid were negative. Although no pathogen was detected, the patient was empirically treated with cefoperazone/sulbactam due to severe dyspnea, but no improvement was observed.

**Table 1:** Parameters of pleural effusion.

	Left	Right
Color Mononuclear macrophages (%) Multinucleated cells (%) Endotheliocytes (%) Rivalta’s test Total protein (g/L) Lactate dehydrogenase (IU/L) Adenosine deaminase (IU/L) Glucose (mmol/L) Carcinoembryonic antigen (μg/L) Culture	slightly turbid yellow 38.7 61.3 1.8 positive 44.1 206 9 4.71 105 (-)	slightly turbid yellow 41.6 58.4 0.6 positive 45.1 147 11 8.23 10 (-)

## The positron emission tomography-computed tomography

**Figure 2:**
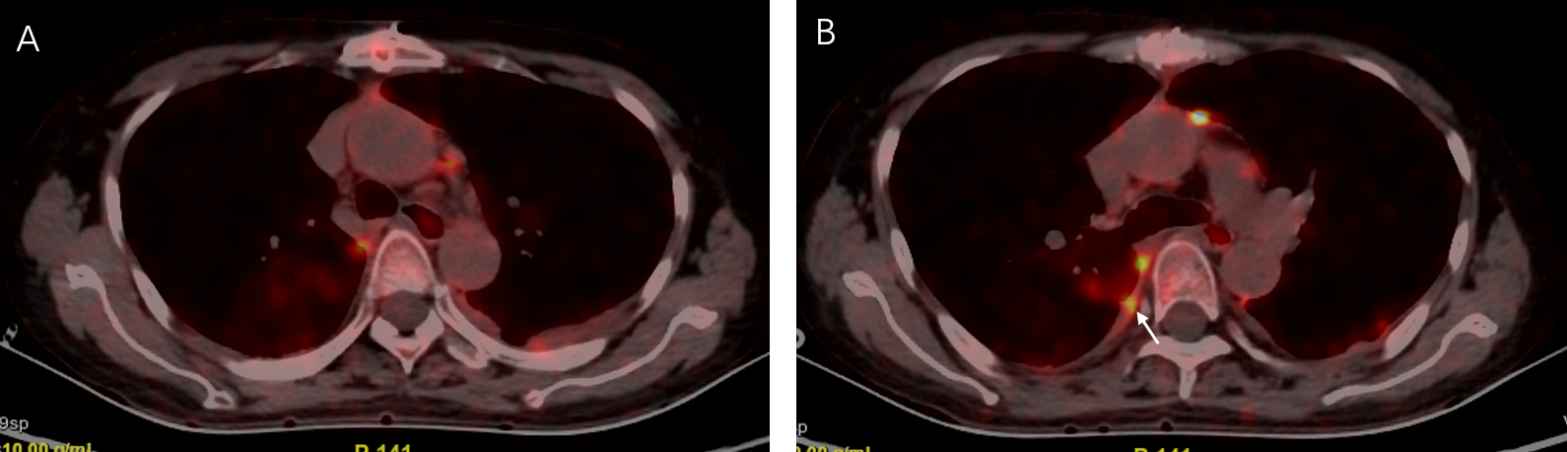
The positron emission tomography was performed 13 days later and showed multiple pleural thickening **(A, B)** and metabolic activity was found bilaterally with a maximum standard uptake value of 12.5 **(B)**, arrow.

The patient was referred to our respiratory department for unexplained pleural effusion and dyspnea. In view of the patient’s history, we believed that both pleural metastases and pleuro-specific infection should be considered. PETCT was performed and showed multiple pleural thickening, and metabolic activity was found bilaterally with a maximum standard uptake value of 12.5 (**Figure 2**).

## Pleural biopsy

**Figure 3:**
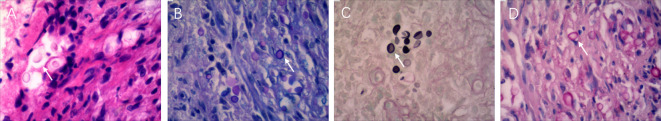
Pathological examination of pleural tissue revealed fungal bodies, which were numerous yeastlike organisms surrounded by mucinous capsules and were between mesothelial cells and histiocytes **(A–D)**, white arrow. Stained by hematoxylin and eosin **(A)**, AB-PAS **(B)**, hexamine silver **(C)**, and mucicarmine **(D)**.

Right pleural biopsy was performed. Pathological examination revealed fungal bodies were between mesothelial cells and histiocytes, which were numerous yeast-like organisms surrounded by mucinous capsules. *Cryptococcus* was highly suspected by Alcian Blue–Periodic Acid-Shiff (AB-PAS), hexamine silver, and mucicarmine staining of pleural tissue (**Figure 3**). Meanwhile, the mNGS technology was performed on pleural tissue and *Cryptococcus neoformans* was detected with a species-specific read number (SSRN) of 4 (https://www.ebi.ac.uk/ena/browser/, accession number PRJEB64498). Ultimately, a diagnosis of pleural cryptococcosis was established in this patient, although there was no exposure factor for *Cryptococcus*.

## Treatment and follow-up

**Figure 4:**
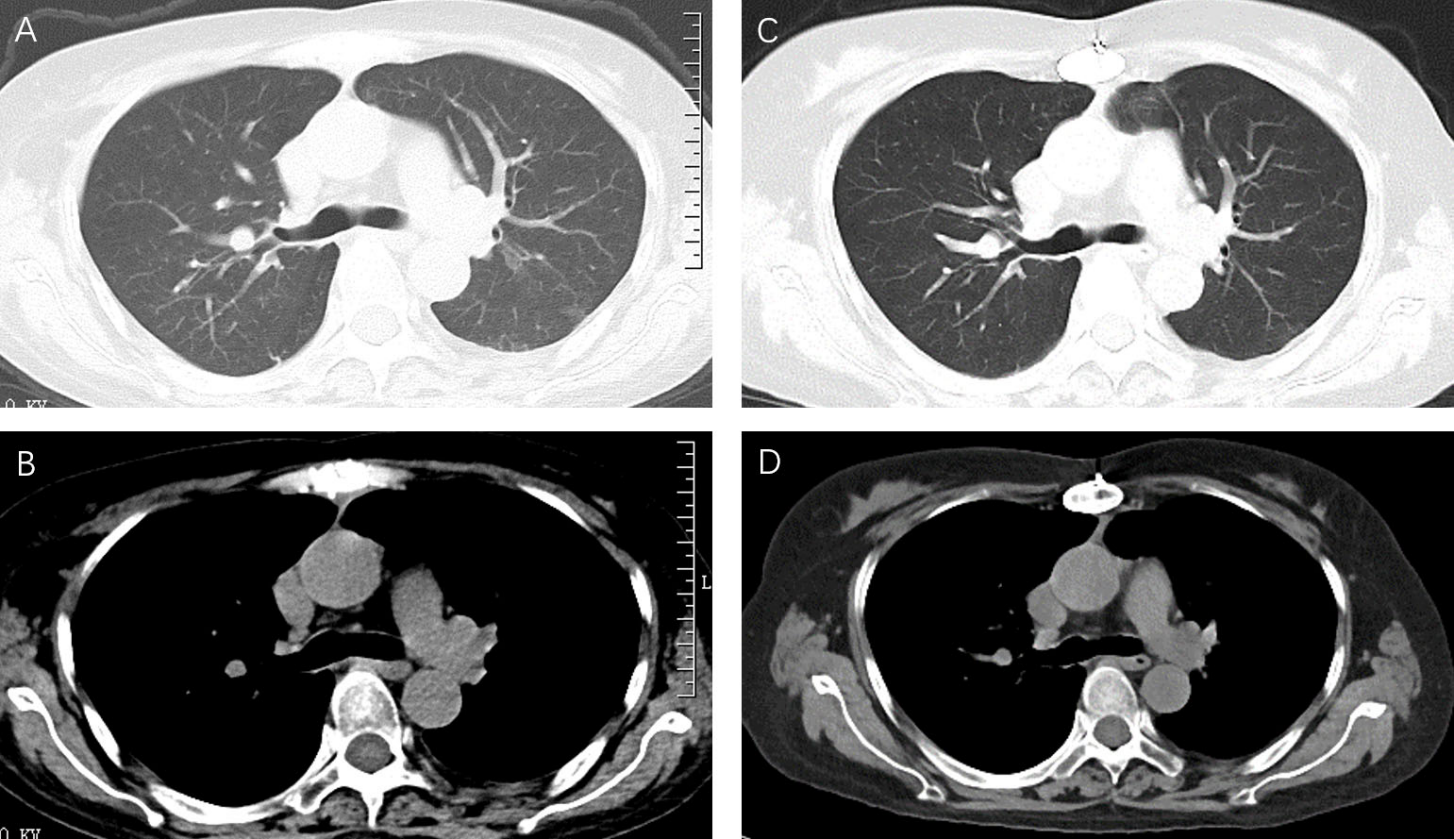
A reexamination of chest CT after 2 months of amphotericin B and fluconazole therapy revealed no pleural effusion **(A, B)**. Follow-up chest CT showed that the patient was still free of pleural effusion 1 year later **(C, D)**.

Prior to treatment, a lumbar puncture was done to rule out central nervous system infection. A therapy of amphotericin B was given. Then, the volume of pleural effusion gradually decreased and the reexamination of thoracic B ultrasound showed no pleural effusion after 1 week. Amphotericin B was discontinued after the full dose and then fluconazole was administered orally for 2 months. The follow-up chest CT revealed no pleural effusion, and the healthy status of the patient was maintained 1 year later (**Figure 4**).

## Discussion

*Cryptococcus* is a rare opportunistic pathogenic fungus found in the environment in association with soil, pigeon guano, and trees. It was first described as a human pathogen by pathologist Otto Busse and surgeon Abraham Buschke in 1894 when they isolated a “*Saccharomyces*- like” organism from a bone infection in a young woman and was finally named *Cryptococcus neoformans* by Jean-Paul Vuillemin in 1901 (Srikanta et al., 2014). The current classification defines two species: *C. neoformans* and *C. gattii. C. neoformans* has a global distribution and is closely associated with avian habitats, while most *C. gattii* strains have been isolated from North America and Australia, and are commonly identified in Eucalyptus trees in the environment (Ellis and Pfeiffer, 1990). The majority of cryptococcosis is caused by *C. neoformans*, whereas there have been several cases of *C. gattii* infection reported since 2012 in China (Fang et al., 2015).

*C. neoformans* can differentiate into several morphological forms including yeast, chlamydospores, pseudohyphae, hyphae, and basidiospores during the sexual reproduction process. Interestingly, *Cryptococcus* generally exists in the yeast form during infection, and formation of hyphae or pseudohyphae, however, has been observed only occasionally (Zhao et al., 2019). This was also confirmed in our case, where yeast-like organisms were found in the pleural tissue.

Exposure through inhalation of dried yeast cells or basidiospores is common. After inhalation, immune cells recognize the cell wall components of the *Cryptococcus* through pattern-recognition receptors. This recognition triggers an innate immune response, which includes phagocytosis by alveolar macrophages (Ristow and Davis, 2021). Since *Cryptococci* are able to survive intracellularly following phagocytosis, they can evade effective immune responses and reside latently in immunocompetent hosts (Wake et al., 2023). When the host immune function declines, cryptococcosis can occur due to key virulence factors such as a polysaccharide capsule, cell wall melanin, and proliferation at human body temperature (Mayer and Kronstad, 2020).

*Cryptococcus* can infect any tissue or organ of the human body, in which the most common site is the central nervous system, followed by the lung and skin. Pleural effusion is a rare manifestation of cryptococcosis and isolated pleural cryptococcosis is extremely rare. Pleural effusion associated with *Cryptococcus* was presented for the first time by W Warr et al. in a 1968 review (Warr et al., 1968). To our knowledge, there are only 14 cases of cryptococcal pleuritis with pleural effusion as the only clinical presentation so far, and most of them were unilateral pleural effusion (Wang et al., 2019). One study shows that pleural effusion was more common in severe immunodeficiency people compared to immunocompetent people (Wang et al., 2021). In our report, the patient had been immunosuppressed with a CD4+ T-cell count of 197.25/μL due to long-term use of corticosteroids (administration of steroids for more than 3 months) and immunosuppressive agents. However, the only clinical manifestation associated with cryptococcosis in this patient was bilateral pleural effusions, which were confirmed as pleural cryptococcosis through histopathology and tissue metagenomic next-generation sequencing. Although we performed a pleural biopsy on only one side, the bilateral effusions resolved after anticryptococcal therapy. The reason for isolated pleural involvement in cryptococcosis is unknown and most of the studies on pleural cryptococcosis are in the form of case reports because of its low incidence.

The diagnosis of cryptococcosis is graded according to probability (probable or confirmed) due to the lack of typical clinical symptoms and the variable sensitivity and specificity of radiologic and mycologic tests (Donnelly et al., 2020). Serum cryptococcal antigen testing has superior sensitivity and specificity in the diagnosis of cryptococci and is the more commonly used noninvasive test. It has been reported in the literature that in cryptococcosis patients with HIV, sensitivity and specificity of serum CrAg were 99.8% (88.4–100) and 95.2% (88.7–98), respectively (Temfack et al., 2021), while in patients without HIV infection, CrAg positivity can be as high as 85.1% (Coussement et al., 2023). G test is usually negative in *Cryptococcus* patients due to the low content of 1, 3-β-D glucan in the *Cryptococcus* cell wall and the thick outer layer of capsular polysaccharides. GM test positivity is also low and false-positive results may occur. CRP and PCT may be elevated in disseminated cryptococcosis, but in isolated lung cryptococcosis, the proportion of patients who had a raised blood CRP level (≥ 10 mg/L) was low (Takazono et al., 2019). In our report, the serum CRP, PCT, G test, and GM test of the patient were negative, which was consistent with previous reports and may be related to cryptococcosis limited to the pleura.

Although serological tests have a good diagnostic value, the criterion for proven cryptococcosis is the histopathologic, cytopathologic, or direct microscopic examination of a specimen obtained by needle aspiration or biopsy from a normally sterile site, which shows yeast cells, or the recovery of a yeast by culture of a sample obtained by a sterile procedure from a normally sterile site that shows a clinical or radiological abnormality consistent with an infectious disease process (Donnelly et al., 2020). Some reports describe an exudative effusion with a predominance of lymphocytes and detection of *C. neoformans* via either pleural fluid culture or antigen detection (Longhitano et al., 2022). However, low pathogen numbers within pleural effusion samples may result in negative culture detection, and the pleural effusion may be nonspecific (Chen et al., 2015). Therefore, it is easy to delay the diagnosis when the patient starts with isolated pleural effusion, especially when the patient has concurrent tumor, which was misdiagnosed as malignant pleural effusion. Diagnostic delays can have significant repercussions including risk of mortality and increase the economic burden (Kabir and Cunningham, 2022). Therefore, early diagnosis is very important. As for our case, when the pleural effusion recurred, a pleural biopsy was performed to establish the correct diagnosis, and the patient recovered well after prompt treatment with amphotericin B and fluconazole.

## Conclusion

Cryptococcosis, manifested by simple pleural effusion, is extremely rare, but when repeated pleural effusion occurs in immunocompromised patients with or in patients with malignant tumors, the possibility of cryptococcosis should be treated with high vigilance and pleural biopsy is recommended if necessary in order to confirm the diagnosis.

## Data availability statement

The data presented in the study are deposited in the ENA repository (https://www.ebi.ac.uk/ena/browser/), accession number PRJEB64498.

## Ethics statement

The studies involving humans were approved by The First Affiliated Hospital of Zhengzhou University’s institutional ethics committee. The studies were conducted in accordance with the local legislation and institutional requirements. The participants provided their written informed consent to participate in this study. Written informed consent was obtained from the individual(s) for the publication of any potentially identifiable images or data included in this article.

## Author contributions

HJ: Data curation, Formal Analysis, Writing – original draft. HL: Formal Analysis, Project administration, Writing – original draft. MT: Data curation, Software, Writing – review & editing. YW: Formal Analysis, Investigation, Writing – review & editing. XH: Methodology, Writing – review & editing. GZ: Conceptualization, Funding acquisition, Writing – review & editing.
